# Silicon attenuates aluminum toxicity in sugarcane plants by modifying growth, roots morphoanatomy, photosynthetic pigments, and gas exchange parameters

**DOI:** 10.1038/s41598-024-53537-8

**Published:** 2024-02-27

**Authors:** Gilmar da Silveira Sousa Junior, Alexander Calero Hurtado, Rita de Cassia Alves, Eduardo Custodio Gasparino, Durvalina Maria Mathias dos Santos

**Affiliations:** 1 Department of Biology Applied to Agriculture, School of Agricultural and Veterinarian Sciences, São Paulo State University (UNESP), Via de acesso Prof. Paulo Donato Castellane s/n, Jaboticabal, São Paulo 14884-900 Brazil; 2 Department of Agricultural Production Sciences-Soil and Fertilizer Sector. School of Agricultural and Veterinarian Sciences, São Paulo State University (UNESP), Via de acesso Prof. Paulo Donato Castellane s/n, Jaboticabal, São Paulo 14884-900 Brazil; 3 University of Sancti Spiritus “Jose Marti Perez” (UNISS), Comandante Fajardo, s/n, Olivos 2, 60100 Sancti Spiritus, Cuba

**Keywords:** Plant development, Plant physiology, Plant stress responses

## Abstract

Aluminum (Al) inhibits growth and limits plant productivity in acidic soils. An important strategy to increase Al tolerance is the use of silicon (Si) nutrition. Thus, the aim of this study was to evaluate the interactive role of Si in increasing the growth, physiological and morphoanatomy responses of sugarcane plants under Al toxicity. A 4 × 2 factorial scheme in a completely randomized design was used to study the impact of Si (2 mM) on attenuating Al toxicity (0, 10, 15 and 20 mg L^−1^, as Al_2_(SO_4_)_3_·18H_2_O) in sugarcane seedlings. After 45 days, Al toxicity affected sugarcane growth by increasing Al uptake and accumulation, modifying root growth, thickness, and morphoanatomy, and decreasing pigment content, gas exchange parameters, and the number of adaxial and abaxial stomata. However, Si attenuated Al toxicity in the sugarcane seedlings by limiting Al uptake and transport to the shoots, causing positive changes in root morphoanatomy, higher pigment content, improving gas exchange parameters, thereby increased growth. Furthermore, cultivar ‘CTC9003’ showed beneficial impacts from Si supplementation than ‘CTC9002’, especially under Al toxicity. The findings of this study suggest that Si plays a notable role in improving anatomical and physiological aspects, particularly the growth of sugarcane seedlings under Al toxicity.

## Introduction

Sugarcane production has high social and environmental relevance. These factors led, from the end of the twentieth century, to the expansion of cultivation to the Midwest region of Brazil, which is characterized by acidic soils (pH ≤ 5.5), comprising Brazil’s potentially arable land^1^. In acid soil, toxic levels of Al, manganese (Mn), iron (Fe), and phosphorus (P) are the major limiting nutrient on acid soils^2,3^. Opportunely, Al is mostly bounded in non-phytotoxic forms^3,4^, however, under acidic soil conditions (pH ≤ 5.5), Al is solubilized into aluminum ion (Al^3+^) and became biologically available^5^. Leaching of alkaline cations, Ca^2+^ and K^+^, from the soil, acidic rainfall, and acidic mine drainage are among the major causes for soil acidification and generation of the phytotoxic hazards of Al^6^. The first symptom of Al phytotoxic is a quick inhibition of root growth, which lead to low formation of root hairs, reduced permeability of root cells and greater thickening of root apexes by directing multiple key cellular components, resulting in a range of adverse effects such as reactive oxygen species (ROS) production, plasma membrane damage, intracellular transport disruptions, and Ca^2+^ homeostasis disequilibrium^7,8^. In addition, Al toxicity limits water and mineral nutrient uptake, which further leads to sizable losses of crop growth and yield^9,10^. Therefore, Al phytotoxicity inhibits leaf growth and affects secondary metabolites such as plant pigments, chlorophylls (Chls *a* and Chls *b*), carotenoids (Cars) and anthocyanins (Anths), amongst others^11^. Several reports have provided indications that Al toxicity reduces photosynthetic rate (PN), stomatal conductance (*g*s) and leaf transpiration rate (*E*) in plants^12–15^. Plants exposed to Al toxicity effects undergo changes in their cell organelles; e.g., breakdown of free cytosolic^16^ Ca^2+^, callose deposition in plasmodesmata^17^, inhibition of respiration in mitochondria^18^, and decreased uptake of macro and micronutrients^8,10^.

One alternative method involving the mitigation of the negative effects of stresses can be the use of Si^8,19^. The beneficial effects of Si in attenuating the adverse effects of Al toxicity have been reported in various Si-accumulating plants such as, sorghum^20^, sugarcane^1,8,10^, barley^21,22^, and wheat^2^. Si affects entry and detoxification of metal ions in the plant body. The immobilization of toxic metals is correlated with Si deposition in the cell wall of roots. Si reduces the binding site of Al by increasing the wall-bound form of Si, thereby reducing the ability of Al to bind to the cell wall, consequently leading to detoxification of Al and attenuation of root growth inhibition^23,24^.

The benefits of Si in combating heavy metals, especially Al toxicity, are mainly related to the formation of protective and regulatory complexes, which cause changes in soil physical conditions, such as changes in soil pH, metal speciation or decreased metal availability (loids) due to co-precipitation with Si^25^. The decrease in protoplast/apoplast ratio by Si treatment may be a crucial mechanism to decrease the toxicity of excess metals (loids) in metabolically important parts of cells, such as the cytoplasm and all organelles^12^. Also, Si decreased the intergranular space and increased the grana density in chloroplasts^26^. Si supply also reverses double membrane disintegration and abnormality ridge expansion in mitochondria^6,27^.

Si application is important in mitigating Al toxicity by minimizing transpiration and maximizing photosynthesis^28,29^, increasing phenolic compounds, especially lignin^10,21,22^, enhancing enzyme activity^6,30^, and macros- and micronutrients uptake^6,10,12^. Several studies with various species found that, Si supplementation enhances plant responses to the toxic effects of Al in plants such as wheat^31^, maize^32^, rice^33^, and barley^21,22^.

Sugarcane was selected in this study due to its hyperaccumulation of Si^8,34^, which is suitable for studying the mechanisms of Si-mediated Al tolerance. Currently, most studies on the amelioration of Al toxicity by Si are based on growth and physiological levels^22,35^, while the role of Si in root morphoanatomy under Al stress is still limited. Therefore, the objective of this study was to evaluate the interactive role of Si in increasing the growth, physiological and morphoanatomy responses of sugarcane plants under Al toxicity.

## Results

### Growth parameters and relationship between Al and Si concentrations

**Figure 1:**
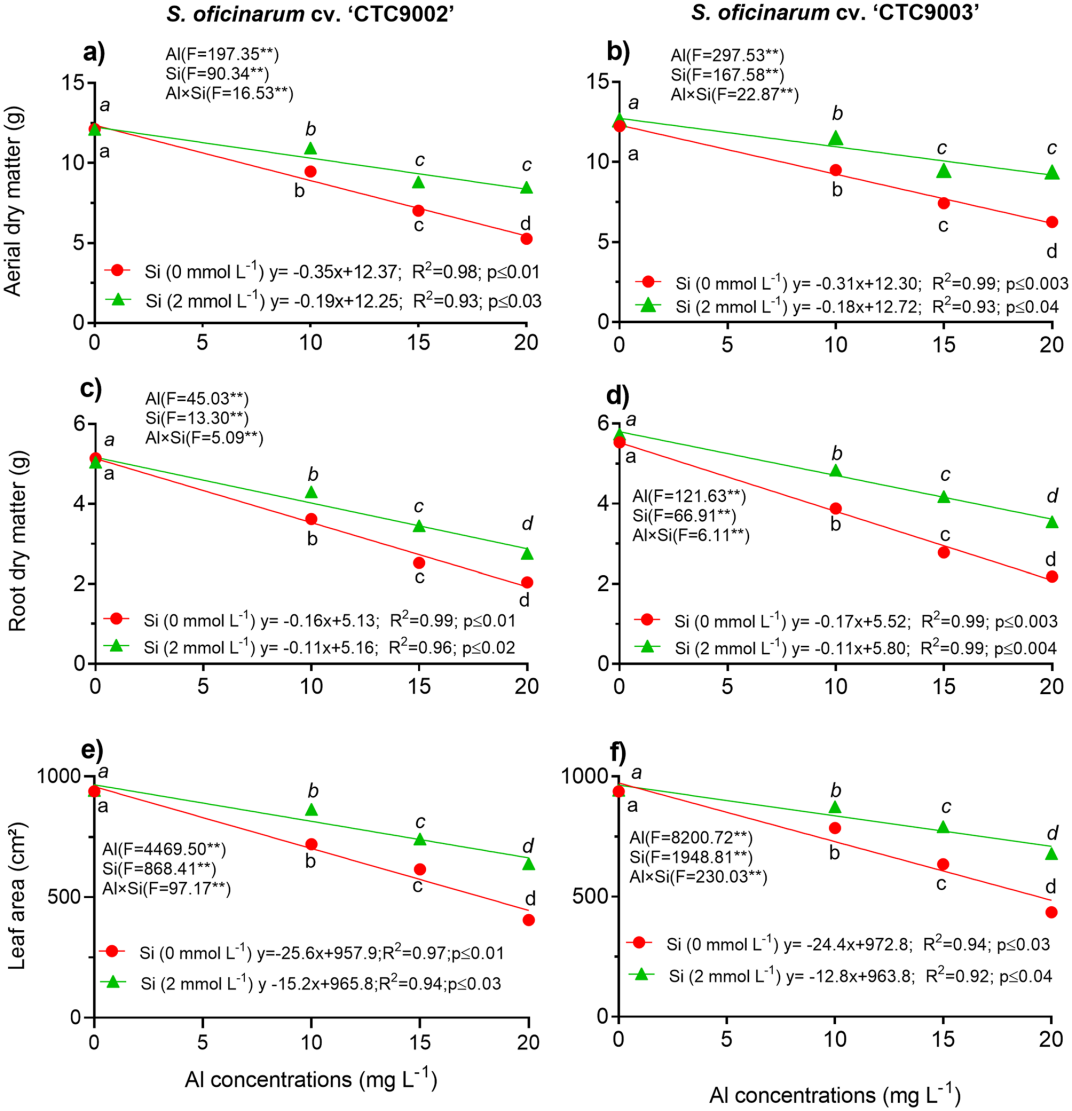
Aerial dry mass cv ‘CTC9002’ (**a**) and cv ‘CTC9003’ (**b**), root dry mass cv ‘CTC9002’ (**c**) and cv ‘CTC9003’ (**d**) and leaf area cv ‘CTC9002’ (**e**) and cv ‘CTC9003’ (**f**). Treatments: without Al stress and Si addition (0Al + Si); with Al stress (10 mg L^−1^) and Si addition (2 mmol L^−1^) (10Al + Si); with Al stress (15 mg L^−1^) and Si addition (2 mmol L^−1^) (15Al + Si) and Al stress (20 mg L^−1^) and Si addition (2 mmol L^−1^) (20Al + Si). Data are the means (n = 4) ± standard deviation (SD). Different normal lowercase letters (e.g., a, b, c, d) indicate significant differences among Al concentrations under non-Si addition, and different italic lowercase letters (e.g., *a*, *b*, *c*) indicate significant differences among Al concentrations treatments under Si addition (2 mmol L^−1^), according to the Tukey test and F values from the ANOVA (p < 0.05); **: indicates that F is significant at p < 0.01.

A two way-ANOVA showed significant interaction (p < 0.001) between Al and Si on aerial dry matter (ADM), root dry matter (RDM) and leaf area (LA) of both sugarcane cultivars (Fig. 1a–f; and Tables S1–S3). A linear decrease in ADM, RDM, and LA of both sugarcane cultivar was observed with increasing Al treatments, but this effect was partiality reversed with the addition of Si in the growth medium (Fig. 1a–f). Under non-Al stress conditions, the ADM production was significantly reduced under 10, 15 and 20 mg L^−1^ of Al in the cv. ‘CTC9002’ by 22, 42, and 57% and in the cv. ‘CTC9003’ by 23, 39, and 50%, respectively, compared to the non-Al stress treatments (Fig. 1a,b). Nevertheless, the ADM was increased under Al stress and Si addition in the cv. ‘CTC9002’ by 15, 25 and 61% and in the cv. ‘CTC9003’ by 10, 27, and 50%, under 10, 15 and 20 mg L^−1^ of Al, respectively, compared to the Al stress non-Si treatments (p < 0.001) (Fig. 1a,b). Moreover, under 10, 15 and 20 mg L^−1^ of Al, the RDM was increased significantly (p < 0.001) under Si treatments in the cv. ‘CTC9002’ by 20, 29 and 15% and by 24, 50 and 62% in the cv. ‘CTC9003’, respectively; as compared to the non-Si treatments (Fig. 1c,d). In addition, the LA under Si addition was higher in the cv. ‘CTC9002’ by 13, 20 and 33% and in the cv. ‘CTC9003’ by 14, 24 and 28%, respectively, under 10, 15 and 20 mg L^−1^ of Al, and showed significant difference (p < 0.001) in comparison with the non-Si addition (Fig. 1e,f). Furthermore, the cv. 'CTC9003' showed the greatest reduction of the variables analyzed for growth and the supplementation of Si in this cultivar showed a slight mitigation of the deleterious effects caused by Al toxicity in relation to the cv. ‘CTC9002’.

The correlation analysis revealed the significant positive correlations among shoot [Al] and shoot [Si] under non-Si addition in both sugarcane cultivars (Fig. S1a,b). [Al] in shoots increased significantly (p < 0.001) with increasing Al in the growth medium in the cv. ‘CTC9002’ by 18, 24 and 30% and by 16, 13 and 14% cv. ‘CTC9003’ cultivars, under 10, 15 and 20 mg L^−1^) of Al, respectively (Fig. S1a,b). However, a negative correlation analysis was found among shoot [Al] with shoot [Si] in both sugarcane cultivar under Si addition (Fig. S1c,d). Shoot [Al] under 10, 15 and 20 mg L^−1^ of Al decreased significantly (p < 0.0001) by Si addition in cv ‘CTC9002’ by 10, 19 and 22%; whereas in the cv. ‘CTC9003’ the decreases were 28, 35, and 30%, respectively, as compared to the non-Si addition (Fig. S1c,d).

### Photosynthetic pigments

**Figure 2:**
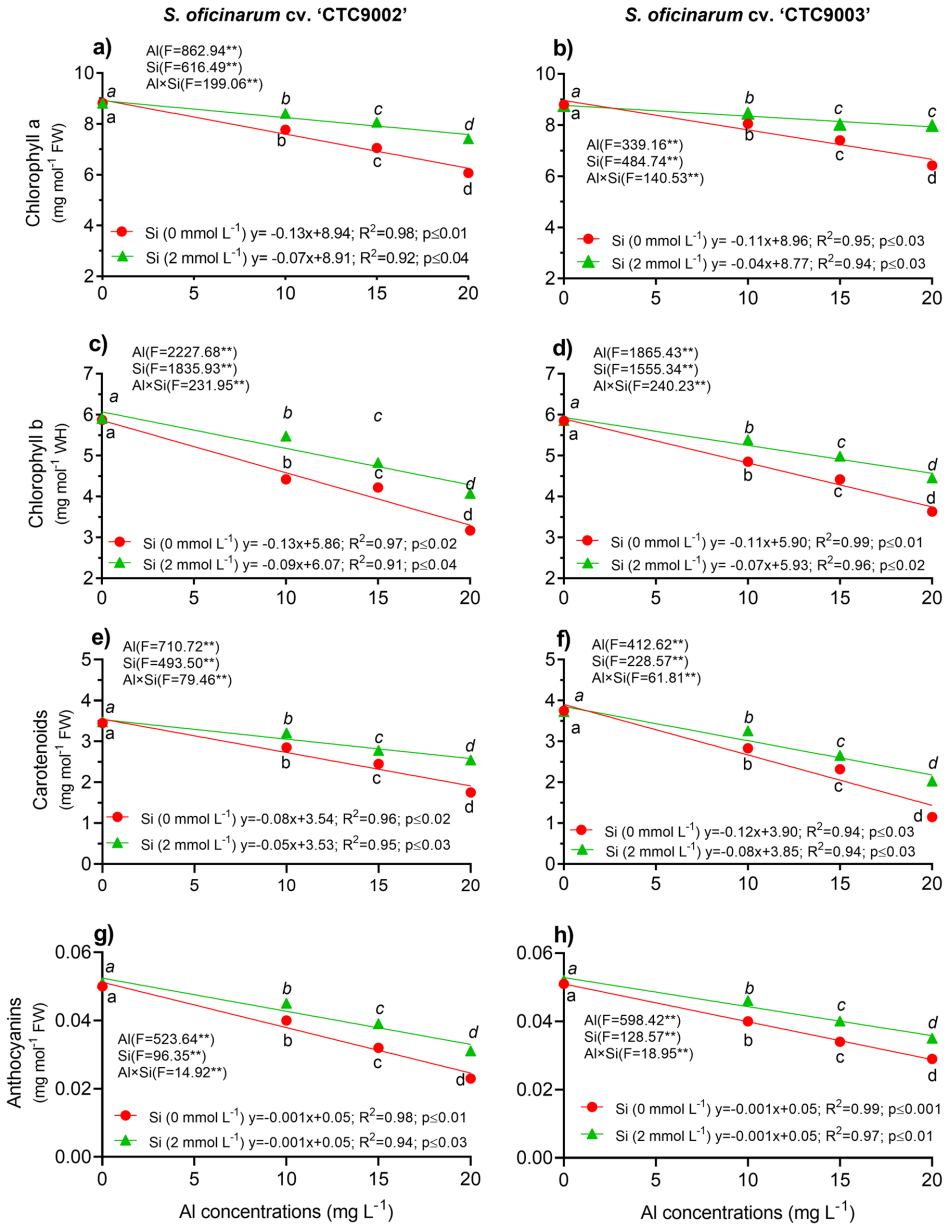
Photosynthetic pigment contents in both sugarcane seedlings. Clhs *a* in cv. ‘CTC9002’ (**a**) and cv. ‘CTC9003’ (**b**); Chls *b* in cv. ‘CTC9002’ (**c**) and cv. ‘CTC9003’ (**d**); Cars in cv. ‘CTC9002’ (**e**) and cv. ‘CTC9003’ (**f**) and Anths in cv. ‘CTC9002’ (**g**) and cv. ‘CTC9003’ (**h**). Treatments and statistics as described in Fig. 1

A bidirectional ANOVA revealed significant interaction (p < 0.001) between Al and Si on plant pigments such as chlorophyll *a* (Chls *a*) chlorophyll *b* (Chls *b*), carotenoids (Cars), and anthocyanins (Anths) (Fig. 2a–h and Tables S4–S7). Increasing Al concentrations caused a linear decrease in Chls *a*, Chls *b*, Cars, and Anths, irrespective of Si addition or non-Si (Fig. 2a–h). Applied Al toxicity significantly decreased the contents of Chls *a* by 17%, Chls *b* by 14%, Cars by 13%, and Anths by 17% in the cv. ‘CTC9002’, whereas in the cv. ‘CTC9003’ these decreases were by 12% in Chls *a*, 13% in Chls *b*, 11% in Cars, and 15% in Anths, respectively, as compared to the non-Al treatment (Fig. 2a–h). However, similar effects were observed in Chls *a* under Si addition and non-Si addition and non-Al stress and 10 mg L^−1^ of Al in both sugarcane cultivars. Moreover, significantly Si addition had greater Chls *a* in the cv. ‘CTC9002’ by 21% and 22%, and in the cv. ‘CTC9003’ by 13% under 15 and 20 mg L^−1^ of Al, respectively as compared to the non-Si addition (Fig. 2a,b). Additionality, the Chls *b* content under Si addition showed similar effects under 10 and 15 mg L^−1^ of Al in both sugarcane cultivar and increasing the Chls *b* by 31% in the cv. ‘CTC9002’ and by 25% in the cv. ‘CTC9003’, while under 20 mg L^−1^ of Al the increased in the cv. ‘CTC9002’ was 25% and 17% in the cv. ‘CTC9003’ compared to the non-Si treatment (Fig. 2c,d).

In both sugarcane cultivars, Si addition reveled similar effects in Cars contents under 15 and 20 mg L^−1^ of Al and showed significant increases by 12 and 38% in the cv. ‘CTC9002’ and by 10 and 22% in the cv. ‘CTC9003’, respectively; whereas in 20 mg L^−1^ of Al, the decreases were 74 and 81% in the ‘CTC9002’ and cv. ‘CTC9003’, respectively, as compared to the non-Si treatment (Fig. 2e,f). Furthermore, the Anths content was significantly increased under Si addition by 25% under 10 and 15 mg L^−1^ of Al and by 39 under 20 mg L^−1^ of Al in the cv. ‘CTC9002’, whereas in the cv. ‘CTC9003’ the increases were 10% under 10 and 15 mg L^−1^ of Al and 19%, respectively, as compared to the non-Si treatment (Fig. 2g,h).

### Gas exchange and stomatal density

**Figure 3:**
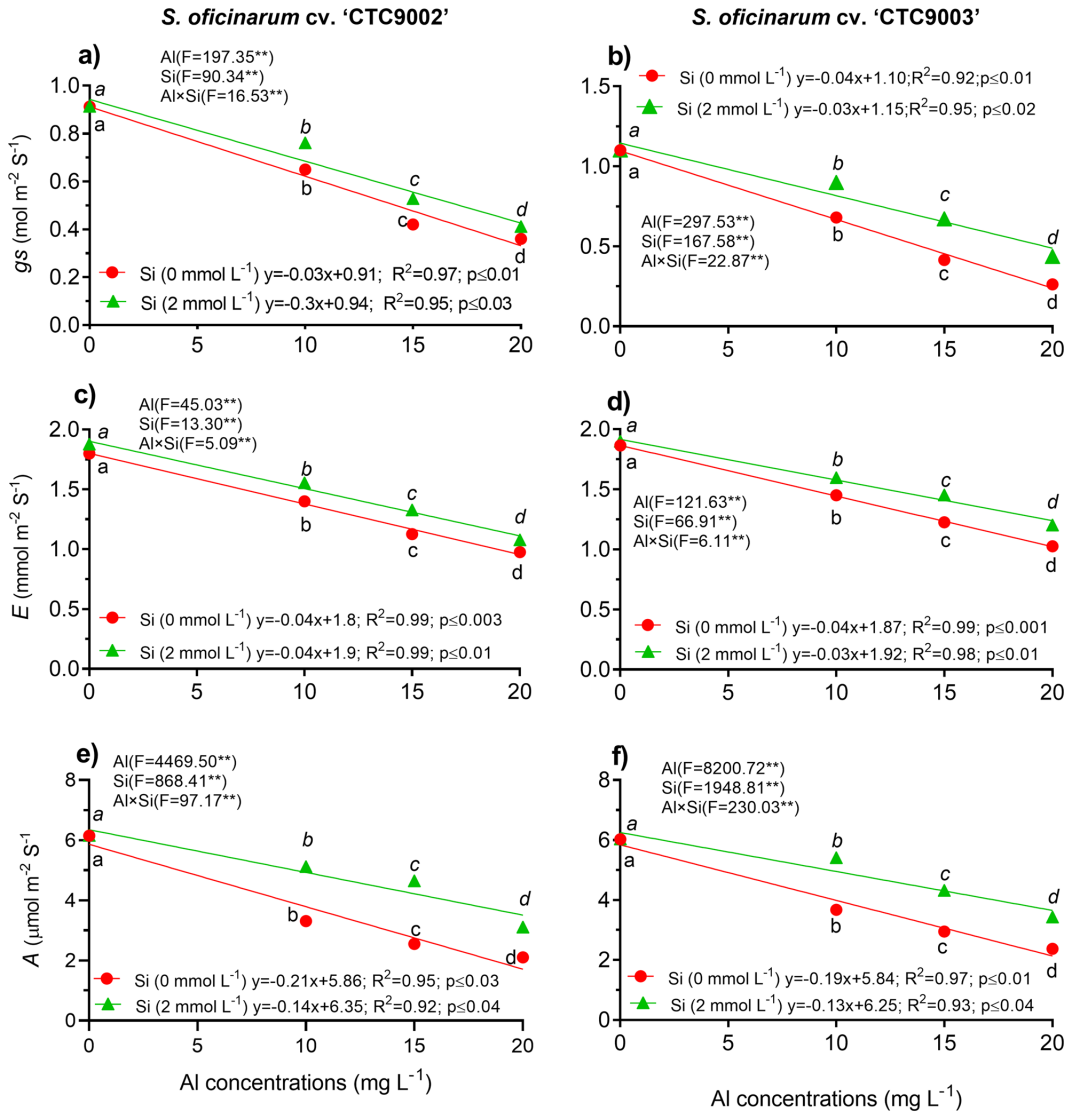
Gas exchanges parameters of both sugarcane seedlings. Stomatal conductance (*g*s) in cv ‘CTC9002’ (**a**) and cv ‘CTC9003’ (**b**); transpiration rate (*E*) in cv. ‘CTC9002’ (**c**) and cv ‘CTC9003’ (**d**); carbon assimilation (*A*) in cv ‘CTC9002’ (**e**) and cv ‘CTC9003’ (**f**). Treatments and statistics as described in Fig. 1

Data regarding stomatal conductance (*gs*), transpiration rate (*E*) and carbon assimilation (*A*) showed significant (p < 0.001) interaction between Al and Si factors in both sugarcane cultivars, according to a two-way ANOVA (Fig. 3a–f; Tables S8–S11). Gas exchange parameters like that *gs*, *E*, and *A* decreasing linearly in both sugarcane cultivar with increasing Al concentrations in the growth medium (Fig. 3a–f). The *gs* in the cv. ‘CTC9002’ and cv. ‘CTC9003’ showed similar effects in the Si treatment under non-Al and 10 mg L^−1^) of Al and was significantly under 15 and 20 mg L^−1^ of Al by 26 and 14% in the cv. ‘CTC9003’ and by 60 and 64% in the ‘CTC9003’ respectively, and showed significant difference (p < 0.001) compared to the non-Si treatment (Fig. 3a,b). Similarly, the *E* was increased by Si treatment under 10, 15 and 20 mg L^−1^ of Al by 11, 18, and 12% in the cv. ‘CTC9002’ and by 10, 18, and 17% in the cv. ‘CTC9003’, respectively, in comparison with the non-Si treatment (p < 0.001) (Fig. 3c,d). Furthermore, the *A* was higher in Si treatment under 10, 15 and 20 mg L^−1^ of Al in the cv. ‘CTC9002’ by 63, 82, and 48% and in the cv. ‘CTC9003’ by 47, 46, and 44%, respectively, and showed significant difference (p < 0.001) compared to the non-Si treatment (p < 0.001) (Fig. 3e,f). We found that the highest gas exchange parameters were recorded in the cv. ‘CTC9003’, while the minimum gas exchange parameters were recorded in the cv. ‘CTC9002’ (p < 0.0001) (Fig. 1a–f).

The number of adaxial stomata (ADS) and number abaxial stomata (ABS) showed significant (p < 0.001) interaction between Al and Si factors in the both sugarcane cultivars, according to the F test (Fig. S2a–d and Tables S11 and S12). The measured ADS and ABS in both sugarcane cultivars decreased linearly with increasing Al concentrations in the growth medium and showed significant difference (p < 0.001) compared to the non-Al treatment (Fig. S2a–d). However, ADS under Si addition was similar under non-Al treatment and 10 mg L^−1^ of Al and was higher by 16% in 10 mg L^−1^ of Al, 22% in 15 mg L^−1^ of Al, and 42% in 20 mg L^−1^ of Al in the cv. ‘CTC9002’; whereas the cv. ‘CTC9003’ the increases were 13, 21, and 44%, respectively, as compared to the non-Si treatment (p < 0.001) (Fig. S2a,b). In addition, the ABS was higher by Si treatment under 10, 15 and 20 mg L^−1^ of Al in the cv. ‘CTC9003’ by 33, 86, and 112% and in the cv. ‘CTC9003’ by 28, 104, and 105%, respectively, and showed significant difference (p < 0.001) compared to the non-Si treatment (p < 0.001) (Fig. S2c,d). Between cultivars, ‘CTC9003’ showed higher values of ADS and ABS. Thus, the beneficial effects of added Si were more prominent in the cv. ‘CTC9003’ than that in cv. ‘CTC9002’, especially in the presence of Al (Fig. S2).

### Morphoanatomy of roots

**Figure 4:**
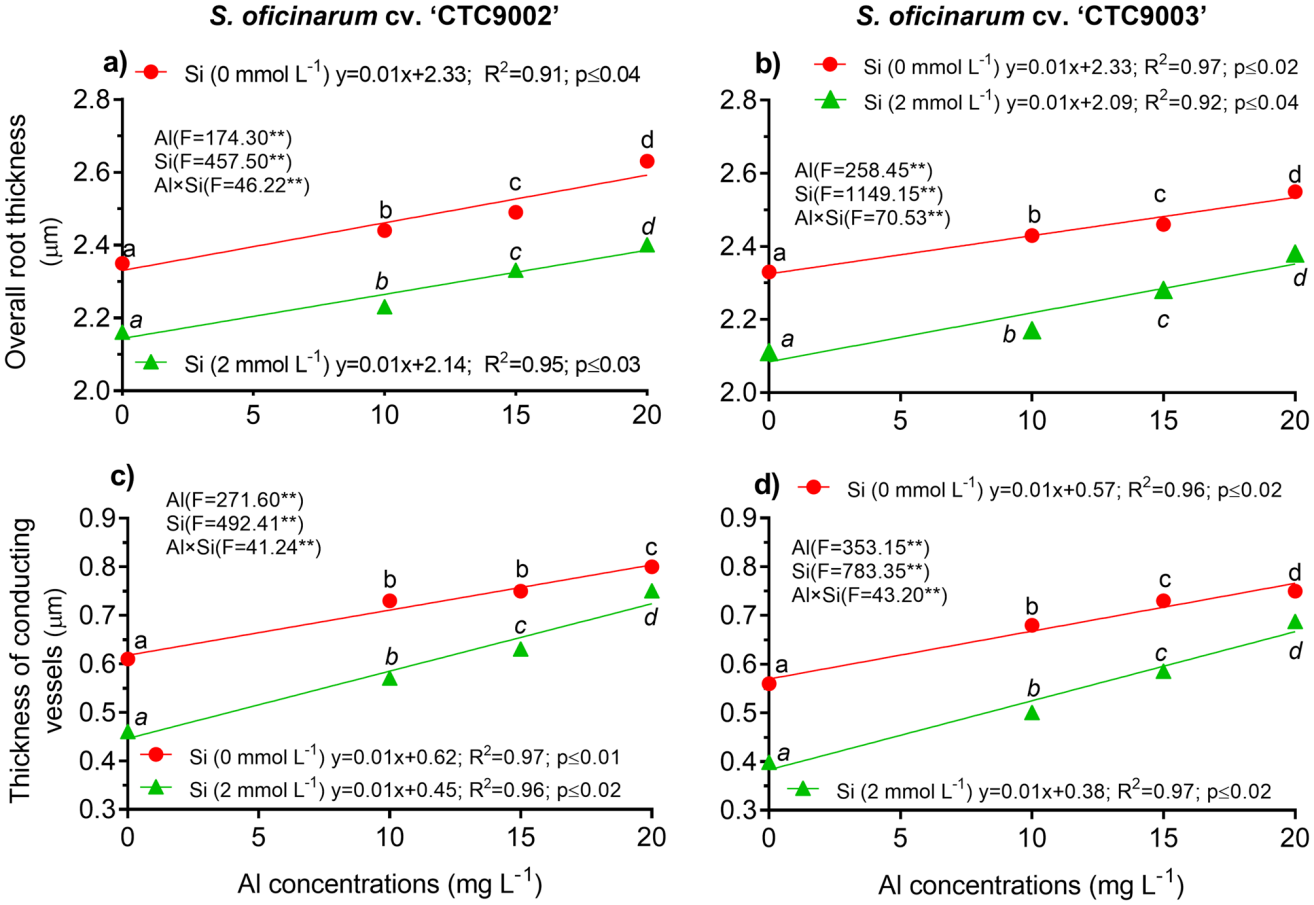
Overall root thickness in cv ‘CTC9002’ (**a**) and in cv ‘CTC9003’ (**b**) and thickness of conducting vessels in cv ‘CTC9002’ (**c**) and in cv ‘CTC9003’ (**d**). Treatments and statistics as described in Fig. 1

**Figure 5:**
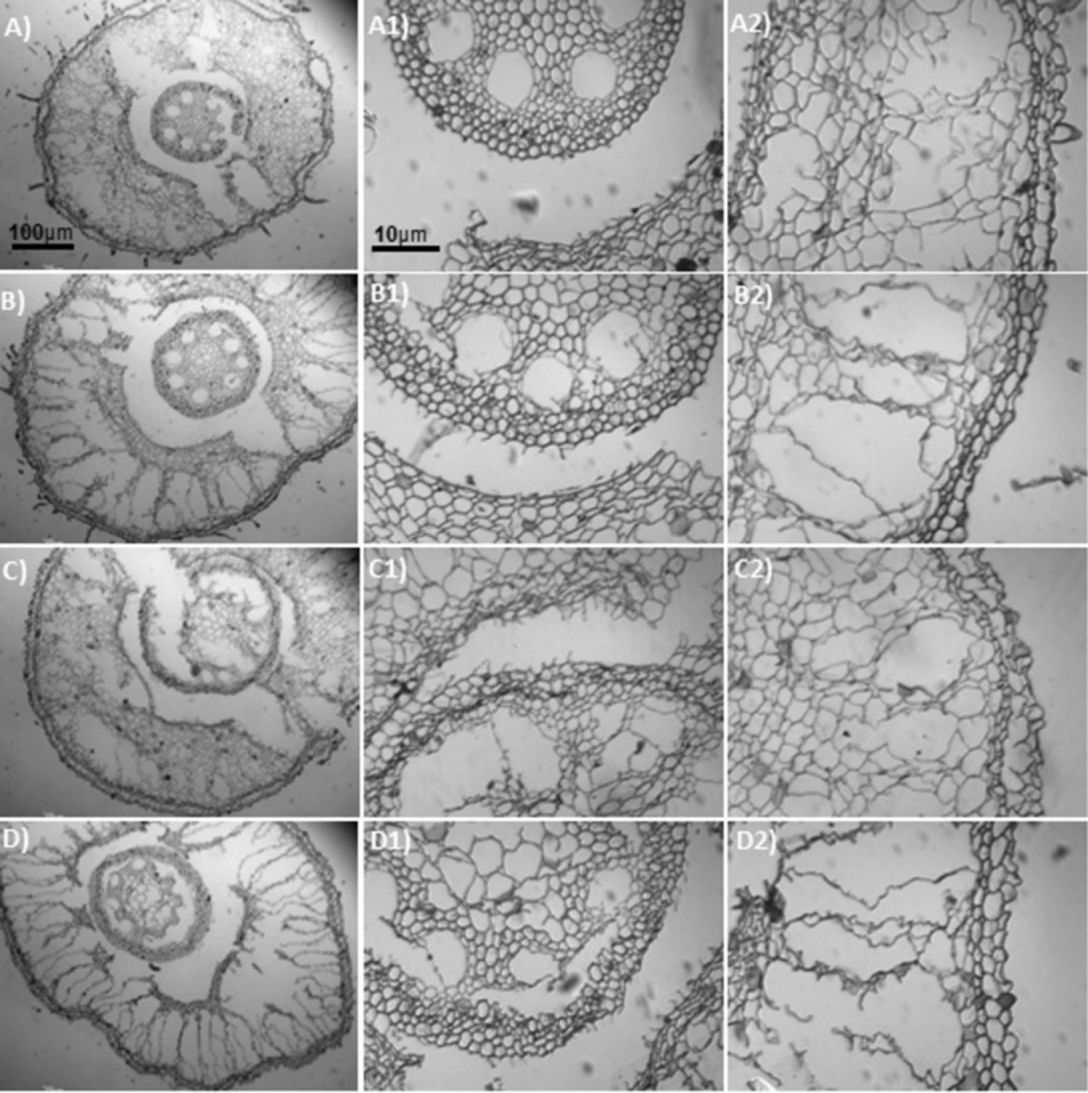
Sections of roots cross (e.g., **A**–**D**), conductive vessels (e.g., **A1**–**D1**) and root thickness (e.g., **A2**–**D2**) of sugarcane cv. ‘CTC9002’ observed in an optical microscope under non-Si addition. Root cross sections under non-Al stress (**A**), under 10 mg L^−1^ of Al (**B**), under 15 mg L^−1^ of Al (**C**), and under 20 mg L^−1^ of Al (**D**) (*first column*). Conductive vessels under non-Al stress (**A1**), under 10 mg L^−1^ of Al (**B1**), under 15 mg L^−1^ of Al (**C1**), and under 20 mg L^−1^ of Al (**D1**) (*second column*). Root thickness under non-Al stress (**A2**), under 10 mg L^−1^ of Al (**B2**); under 15 mg L^−1^ of Al (**C2**), and under 20 mg L^−1^ of Al (**D2**) (*third column*). ((---) 10 µm/100 µm).

**Figure 6:**
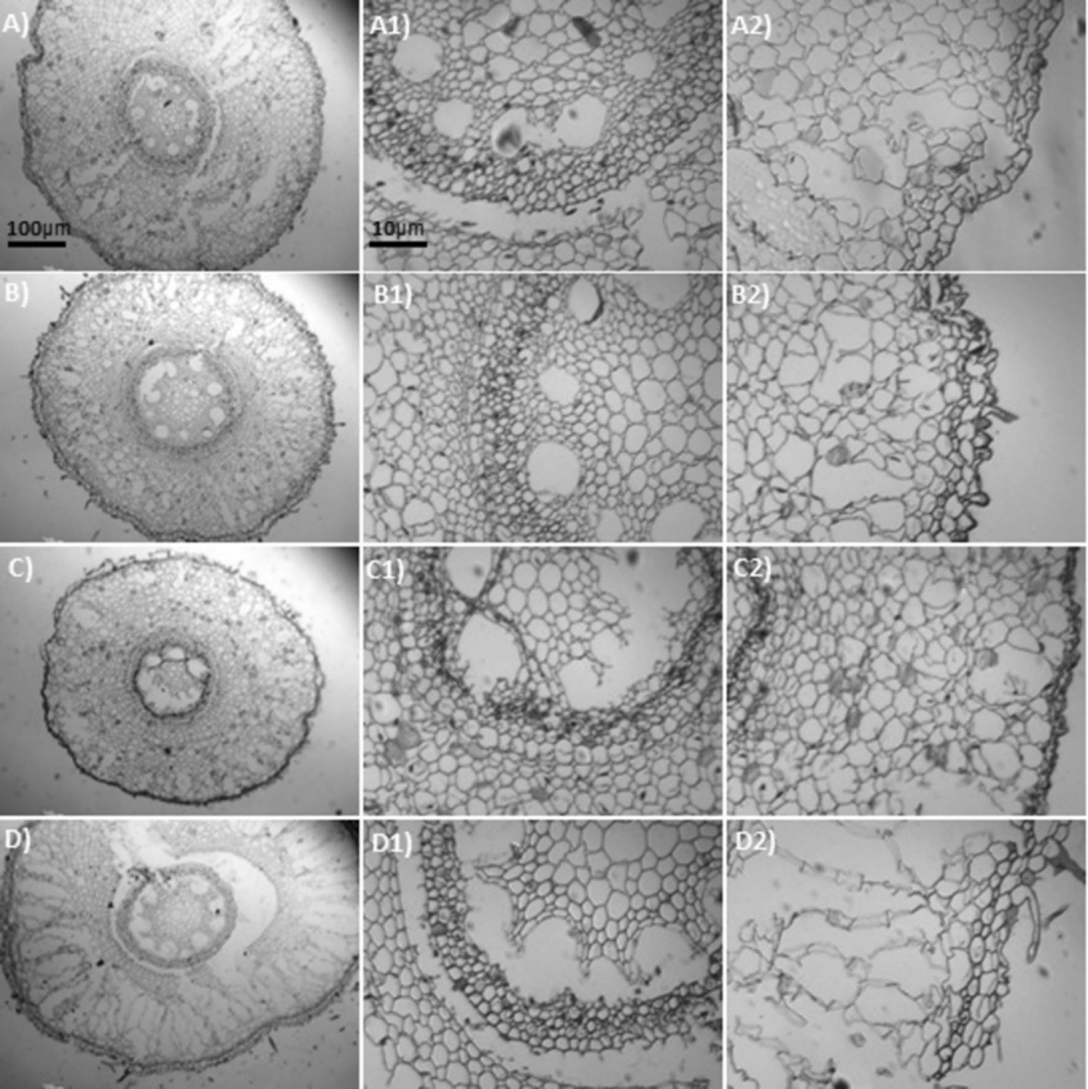
Sections of roots cross (e.g., **A**–**D**), conductive vessels (e.g., **A1**–**D1**) and root thickness (e.g., **A2**–**D2**) of sugarcane cv. ‘CTC9002’ observed in an optical microscope under Si addition (2 mmol L^−1^). Root cross sections under non-Al stress (**A**), under 10 mg L^−1^ of Al (**B**), under 15 mg L^−1^ of Al (**C**), and under 20 mg L^−1^ of Al (**D**) (*first column*). Conductive vessels under non-Al stress (**A1**), under 10 mg L^−1^ of Al (**B1**), under 15 mg L^−1^ of Al (**C1**), and under 20 mg L^−1^ of Al (**D1**) (*second column*). Root thickness under non-Al stress (**A2**), under 10 mg L^−1^ of Al (**B2**); under 15 mg L^−1^ of Al (**C2**), and under 20 mg L^−1^ of Al (**D2**) (*third column*). ((---)10 µm/100 µm).

**Figure 7:**
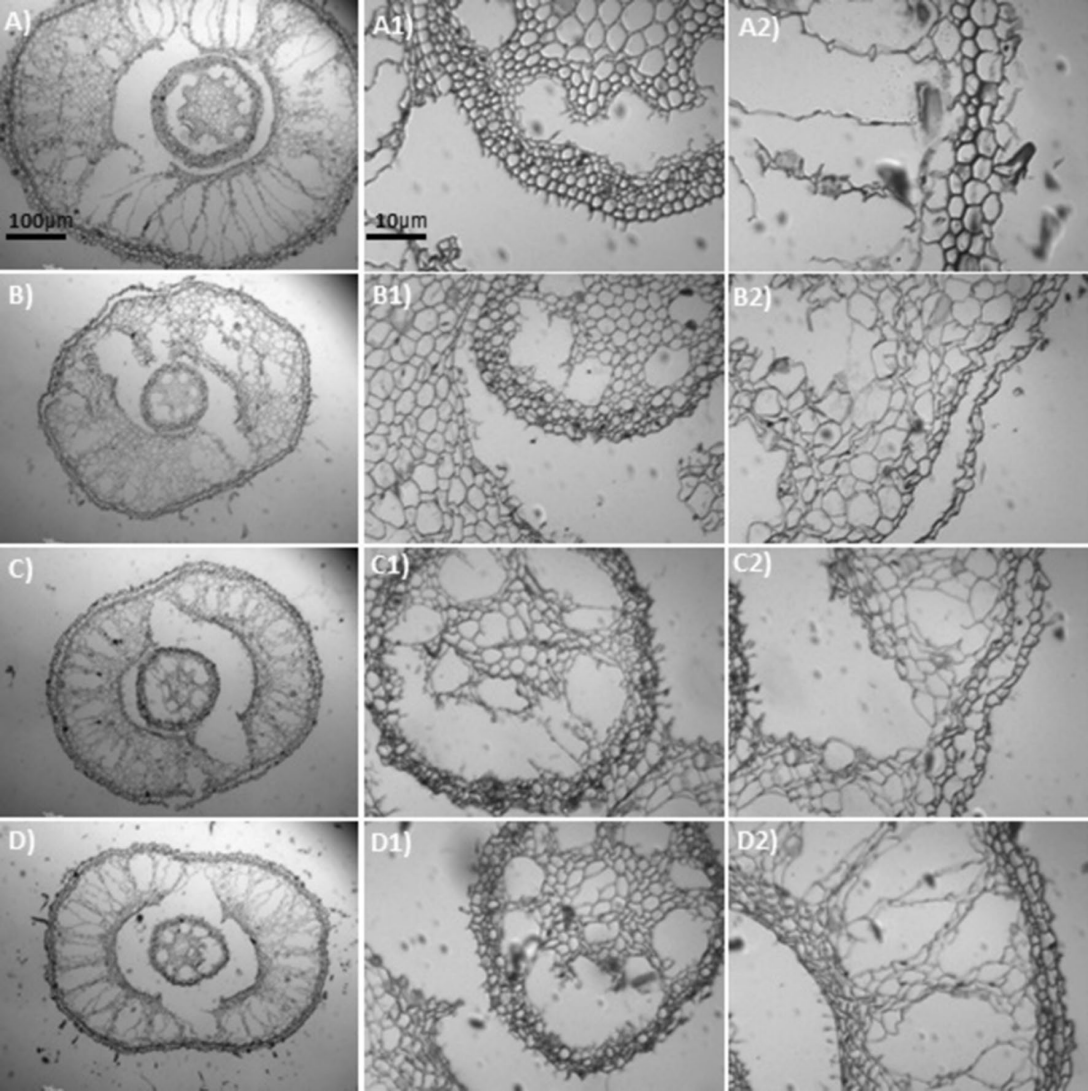
Sections of roots cross (e.g., **A**–**D**), conductive vessels (e.g., **A1**–**D1**) and root thickness (e.g., **A2**–**D2**) of sugarcane cv. ‘CTC9003’ observed in an optical microscope under non-Si addition. Root cross sections under non-Al stress (**A**), under 10 mg L^−1^ of Al (**B**), under 15 mg L^−1^ of Al (**C**), and under 20 mg L^−1^ of Al (**D**) (*first column*). Conductive vessels under non-Al stress (**A1**), under 10 mg L^−1^ of Al (**B1**), under 15 mg L^−1^ of Al (**C1**), and under 20 mg L^−1^ of Al (**D1**) (*second column*). Root thickness under non-Al stress (**A2**), under 10 mg L^−1^ of Al (**B2**); under 15 mg L^−1^ of Al (**C2**), and under 20 mg L^−1^ of Al (**D2**) (*third column*). ((---)10 µm/100 µm).

**Figure 8:**
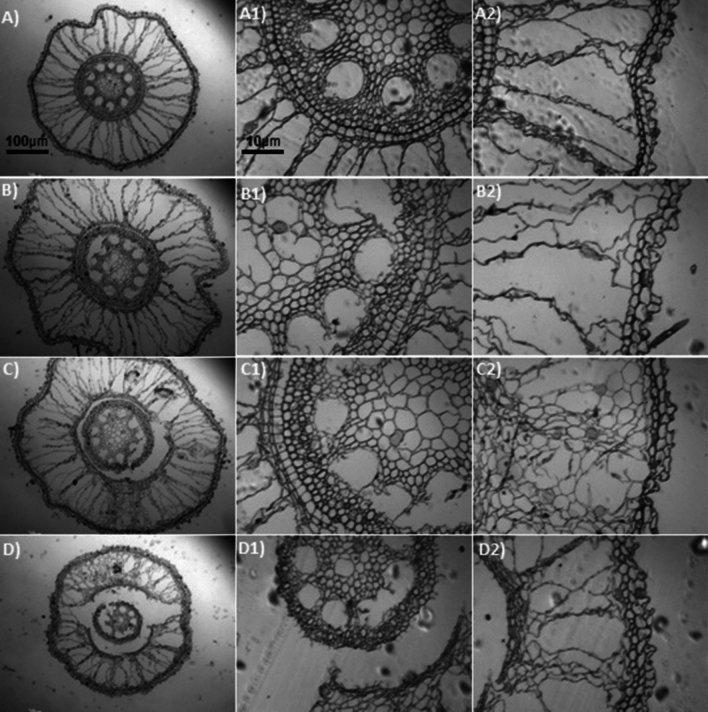
Sections of roots cross (e.g., **A**–**D**), conductive vessels (e.g., **A1**–**D1**) and root thickness (e.g., **A2**–**D2**) of sugarcane cv. ‘CTC9003’ observed in an optical microscope under Si addition (2 mmol L^−1^). Root cross sections under non-Al stress (**A**), under 10 mg L^−1^ of Al (**B**), under 15 mg L^−1^ of Al (**C**), and under 20 mg L^−1^ of Al (**D**) (*first column*). Conductive vessels under non-Al stress (**A1**), under 10 mg L^−1^ of Al (**B1**), under 15 mg L^−1^ of Al (**C1**), and under 20 mg L^−1^ of Al (**D1**) (*second column*). Root thickness under non-Al stress (**A2**), under 10 mg L^−1^ of Al (**B2**); under 15 mg L^−1^ of Al (**C2**), and under 20 mg L^−1^ of Al (**D2**) (*third column*). ((---) 10 µm/100 µm).

A two-way ANOVA showed significant interaction between Al and Si on overall root thickness (ORT) and conducting vessels thickness (CVT) in both sugarcane cultivars (Fig. 4a–d, and Tables S11 and S12). Data regarding ORT and CVT showed a linear increase with increasing Al concentrations in the growth medium, indicating high significant difference (p < 0.0001) between cultivars and among Al stress treatments (Fig. 4a,b). The ORT in the cv. 'CTC9002' under non-Si addition showed similar effect in 10 and 15 mg L^−1^ of Al and increased the ORT by 9% compared to the non-Al addition, but under 20 mg L^−1^ of Al the ORT was increased by 13% (p < 0.0001) (Fig. 4a and more details in Fig. 5A2–D2). Whereas in the cv. ‘CTC9003’, the ORT showed similar effects under 10, 15, and 20 mg L^−1^ of Al and was increased by 10% as compared to the non-Al treatments (p < 0.001) (Fig. 4b and see details in Fig. 7A2–D2). However, Si addition significantly (p < 0.001) decreased ORT by 11, 17, and 13% in the cv. ‘CTC9002’ and by 10, 12, and 15% in the cv. ‘CTC9003’ compared to the non-Si addition (Fig. 4a,b). Moreover, the beneficial effects of added Si were more significant in cultivar ‘CTC9003’ (see Fig. 6A2–D2) than ‘CTC9002’ (see Fig. 8A2–D2), especially under Al toxicity (Fig. 4a,b).

Results indicated that the CVT in both sugarcane cultivars was significantly (p < 0.0001) increased with increasing Al concentration (see details in Figs. 5A1–D1 and 7A1–D1), indicating high significant difference between cultivars and among Al stress treatments (Fig. 4c,d). CVT under Si addition is less compared with non-Si treatments under Al stress. Si addition showed higher CVT under 10, 15, and 20 mg L^−1^ of Al in the cv. ‘CTC9002’ by 13, 37, and 63% and in the cv. 'CTC9003', respectively, as compared to the non-Si treatment (Figs. 4c,d, 6, and 8). Furthermore, the ameliorative effects of added Si were more pronounced in cv. ‘CTC9003’ than cv. ‘CTC9002’, especially under Al toxicity (Fig. 4c,d).

Roots morphoanatomy showed high alterations in both sugarcane cultivars cultivated under Al stress conditions in the absence of Si on root cross (Figs. 5A and 7A). In general, increasing Al concentration generally led to a substantial, significant increase in root cross of cv. ‘CTC9002’ (Fig. 5B–D) and in cv. ‘CTC9003’ (Fig. 7B–D), which was most pronounced in the absence of Al in the growth medium (Figs. 5A and 7A). However, Si addition decrease the root cross depression under 10, 15 and 20 mg L^−1^ of Al in the cv. ‘CTC9002’ (Fig. 6B–D) and in cv, ‘CTC9003’ (Fig. 8B–D) compared to the non-Al addition in the growth medium (Figs. 6A and 8A).

Al stress caused rupture in the region of the ORT (see details for cv. ‘CTC9002’ in Fig. 5B2,C2,D2 and Fig. 6B2,C2,D2, and in cv. ‘CTC9003’, Fig. 7B2,C2,D2 and Fig. 8B2,C2,D2) and CVT (see details for cv. ‘CTC9002’ in Fig. 5B1,C1,D1 and Fig. 6B1,C1,D1, and in cv. ‘CTC9003’, Fig. 7B1,C1,D1 and Fig. 8B1,C1,D1), irrespectively of the non-Si and Si addition in the growth medium. However, Si addition to the growth medium caused a less increases in ORT (see details for cv. ‘CTC9002’ in Fig. 6B2,C2,D2, and in cv. ‘CTC9003’, Fig. 8B2,C2,D2) and CVT (see details for cv. ‘CTC9002’ in Fig. 6B1,C1,D1, and in cv. ‘CTC9003’, Fig. 8B1,C1,D1) as compared with the non-Si treatment (for cv. ‘CTC9002’ in Fig. 5B2,C2,D2 and for cv. ‘CTC9003’ in Fig. 7B1,C1,D1). In addition, Si supplementation allowed greater integrity in the region of the ORT and VCT, and the beneficial effects of added Si were more marked in cv. ‘CTC9003’ (for ORT see Fig. 8A2,B2,C2,D2 and for CVT see Fig. 8B1,C1,D1) as compared to the cv. ‘CTC9002’ (for ORT see Fig. 6A2,B2,C2,D2 and for CVT see Fig. 6B1,C1,D1), especially under Al stress.

## Discussion

In current farming systems, plants are exposed to different stress conditions, which lead to restrict their growth and limiting their productivity. Si plays an important role in plant growth due to its positive impacts on physiological parameters and mechanical strength, thereby increasing plant resistance to abiotic stresses. Several potential mechanisms by which Si can stimulate plant resistance against Al stresses have been reported in the literature, i.e., restriction of root uptake and immobilization of metal(loid)s in the rhizosphere^12^, formation of insoluble complexes between Si and metal(loid)s^36^, influences in oxidative mechanism by modifying the osmolytes accumulation, improves membrane stability, and acts on gene expression^22^, improving nutrient uptake and homeostasis^8^ and stimulation of the production of phenolic compounds with either antioxidant or structural functions^10,21^. In the current study, exogenous Si application exhibited beneficial effects in attenuating the adverse effects of Al in sugarcane seedlings by modifying the root (morphoanatomy) and aerial growth, photosynthetic pigment and gas exchange attributes and decreases Al concentration in shoots. These positive effects of Si on improving growth in sugarcane genotypes under Al stress have been earlier reported^1,8,10^. The improvement of Si in plant growth under Al stress can be explaining due to Si help to preventing the destruction of chlorophyll, enhancing light available for photosynthesis implementation^37^. Another probable mechanism of Si- mediated positive effect on plant growth, such as increased dry mass under Al stress, could be attributed to Si ability to increase water and nutrients uptake and macros- and micronutrients accumulation^10^.

Growth characteristics of both sugarcane cultivars were increased with Si supplementation (Fig. 1a,b,e,f). This fact is in agreement with previous studies, which reported that the first impact of Al phytotoxic are in root growth, resulting in a reduced root system^38^. Also, high Al inhibits roots cell division–elongation, root hair formation, and enhances the development of swollen roots apices^6^. However, in this study significantly promoted the sugarcane growth under Al toxicity may be attributed to Si directly decrease Al uptake and accumulation in shoots (Fig. S1a–d), which help to increases RDM, ORT and CVT (Figs. 1c,d, 4, 5, 6, 7, 8), leading to increase ADM accumulation^10^. Our dates show that Si increased LA in both sugarcane cultivars (Fig. 1e,f). These increases in LA probably occurs by Si increase the ADS and ABS (Fig. S2). These phenomena can be explained in the current study by Si increase the photosynthetic pigment such as Chls *a*, Chls *a*, Cars, and Anths (Fig. 2), leading to increase ADM. These enhancements by Si addition were more effective under 10 mg L^−1^ of Al than other Al levels stress and the cultivar ‘CTC9003’ showed better responses. The Si-mediated beneficial effect on stability of stressed plant cell membranes suggests that Si prevents the deterioration of cell membranes structures and functions, maintaining membrane integrity and functions of stressed plants, improving plant growth and productivity due to the alleviation of stress conditions^19,37^. Additionally, Si impacts on the growth of Al-stressed plants due to its beneficial influence on plant nutrition, antioxidative defense mechanisms, and strengthening of physiological and molecular mechanisms, which leads to increased plant tolerance^10,19,37^. These findings suggest that Si plays an important role in plant growth and development under Al toxicity conditions.

Under Al toxicity conditions, RDM accumulation, ORT, and CVT of both sugarcane cultivars were limited (Figs. 4, 5, 6, 7, 8). However, the effect of Si on the entire root system under longer-term Al stress is unclear. In this study, Si significantly promoted RDM accumulation (Fig. 1c,d). These findings suggested that Si could effectively attenuate Al toxicity in sugarcane. One possible explanation for this fact is due to Si significantly decreased the Al concentration in roots of sugarcane, thereby promoting root growth^8,10^. In addition, Si inhibit roots suberization and Al transport, which lead to restore the roots development, to recuperate the nutrient uptake capacity and to promote the growth of rice^24^. This may be related to the fact that the formation of Al–Si complexes in the root apoplast provides an important barrier to Al penetration in roots^12^. Many studies in the last decades have highlighted the role of Si in ameliorating roots characteristics and growth un plants under Al toxicity^31,32,35,39–42^. The main mechanisms responsible for this action by Si include reducing Al uptake^23,24^. These effects were also evidenced in this study, but Si supplementation in the growth medium reversed the damaging effects in roots of both sugarcane cultivars.

Under Al stress condition, due to the higher presence of Al phytotoxic, OTR and CVT were modifying in response to Al toxicity has been reported for several plant species in rice plants^39^ and maize^43^. This anatomic phenomenon under Al stress has been reported for several plant species^33^. It was reported that Si could mitigate Al stress by improves roots growth characteristics^2,6^. This is the first report to evaluate the beneficial effects of added Si in OTR and CVT under Al stress. Similar findings was obtained with Si addition under Al toxicity, such reduction has been attributed to nuclear damage and vacuolar or mitotic abnormalities in root meristematic cells^39^. Others experimental studies observed significant structural alterations on xylem diameter, mesophyll and epidermis thickness, and the transversal area occupied by collenchyma and midvein treated with Si, which played an important role on the maize tolerance to Al stress^12,44^. In addition, Si increased stomata and root hairs, length of root hairs and leaf epidermal cells, and distorted the structure and integrity of mesophyll cells and phloem^45^. Results showed that Si addition protected rice seedlings against Al toxicity by decreasing Al accumulation and by maintaining level of some mineral elements, and the key structures of leaf and root^36^. However, the attenuation of Al stress by Si may be related to Si function in blocking the intensification of the deleterious effects of Al on the roots, thus improving water uptake and leaves hydration^8,37^. Furthermore, Si promoted the elongation of adventitious roots and the growth of lateral roots by reducing the Al accumulation in roots of rice^45^.

Our data showed that increasing Al concentration in the growth medium increased shoot [Al] in both sugarcane cultivars (Fig. S1). In addition, compared to Al stress, adding Si significantly decreased the shoot [Al] (Fig. S1). These results suggested that Si restricted the Al uptake and transport, thereby reducing the accumulation of Al. Similar findings adding Si decreased the [Al] is by symplastic sap by down-regulating the expression of genes involved in Al uptake and sequestration such as *OsNRAT1* and *OsALS1*^45^. The beneficial impacts of Si in decreasing Al uptake and translocation to the leaves was earlier demonstrated in sugarcane^10^. At the same time, plants need to accumulate Si under Al toxicity so that it can positively influence physiological and antioxidative mechanisms^12,37^. Basically, this relationships can be explained due to the increase in Si concentration, leading to a decrease in the concentration of others elements, including Al^46^. Another probable mechanism of Si in decreasing Al uptake in plants is which Si transfers tolerance to crops, by inactive biological hydroxyaluminosilicates (HAS) complexes can be formed in root epidermis cells^31^, and by formation of compounds that inhibit Al transport in the root-cell symplast^39^, and by formation of chelates as organic acids in the roots and aboveground parts of plant seedlings^47^ or the formation of Al–Si complexes in the external solution^32^ and the regulation of metal transporter genes that decrease the uptake of metalloids^27^. These findings indicate that Si plays an essential role in maintaining low level of Al, which is considered the main mechanism to increasing plant tolerance to Al toxicity.

Syntheses and pigment content are an integral protective mechanism in plants grown under conditions of Al stress. In this study, pigments contents such as Chl *a*, Chl *b*, Cars, and Anths were limited by Al stress (Fig. 2). In addition, compared to Al stress, adding Si significantly increased Chl *a*, Chl *b*, Cars, and Anths in leaf of both sugarcane cultivar. These results suggested that Si play an important role in enhancing plant pigments under Al stress. On the one hand, this could have been the direct consequence of the leaf extension. In addition, Si supplementation prevent chlorophyll destruction, which extends the leaf area, producing more light accessible for photosynthesis^37^. In this study, we demonstrate that Si supplementation improving leaf Chl *a*, Chl *b*, Cars, and Anths and this enhancing may be attributed to that Si is improved leaf area^10,46^. Similar findings with Si supplementation in enhancing photosynthetic pigment biosynthesis and promoting positive impact on photosynthesis were reported in cowpea plants under Al toxicity^40^ and buckwheat^48^. A possible explanation for these increases in photosynthetic pigments by Si in sugarcane under Al toxicity may be related to the important role that Si plays in plant mineral nutrition, especially in increasing the uptake and translocation of Fe and Mg^10^. Similarly, in wheat plants under Al stress the addition of Si increased photosynthetic capacity^49^. Similarly, Si supplementation in rice plants provided better leaf architecture, allowing better light absorb and increasing photosynthetic rate^37^. The beneficial effects of Si on increasing pigment content under Al conditions were reporter in wheat^48^ and watermelon plants^38^. Increasing chlorophyll biosynthesis is another mechanism whereby Si improving K^+^ uptake, increasing chloroplast number per cell, preserved chloroplast ultrastructure, or sustained chlorophyll stability by accelerated ROS-scavenging activity^50^. This study provides the first evidence that Si plays an active role in increasing Anths content in Al-stressed sugarcane plants. These results indicate that plants supplemented by Si might be more productive under adverse environmental conditions, especially under Al toxicity^1^. This increasing in photosynthetic pigment by Si represents an economic strategy for plants to confront with Al stress.

There is no doubt that Al toxicity can inhibit the leaf gas exchange parameters, however, the effect of Al on the leaf gas exchange parameters is not clear. In this study, we found that Al limited the development of leaf gas exchange parameters, including lower *g*s, *E*, and *A* of both cultivars (Fig. 3). On the contrary, Si increased *g*s, *E*, and *A* of both cultivars probably by reducing the Al accumulation in shoots (Fig. S1) and increasing LA^48^. In addition, reduced transpiration is a key mechanism by which Si increases Al tolerance in plants^12,36,41^. In addition, these findings are in line with previous reports, indicating the potential role Si supplementation in attenuating Al stress by promoting the leaf gas exchange parameter in cowpea plants^40^. Additionally, this study reports for the first time the beneficial effects of Si addition on increased ADS and ABS in sugarcane seedlings under Al toxicity (Fig. S2). Here, Al stress caused a reduction in ADS and ABS, but Si supplementation could mitigate Al toxicity by a higher ADS and ABS production, which may have also contributed to a decrease *A*. Similar effects were observed previously in maize plants under water deficit, which allowed better regulation of water transport and transpiration^51^. Our results have exhibited that Si enhances gas exchange response in sugarcane under Al stress. In sugarcane, Si application in the growth medium improves stomatal conductance. Taken together, Si helps sugarcane plants to increase gas exchange parameters, which leads to an improvement in physiological process. Therefore, the results of this study indicate that Si plays an important role in increasing the tolerance of sugarcane plants under Al toxicity by improving physiological processes.

Our results also showed that the interaction between Si and Al stress treatment varied between cultivars of the same species. In the current study the cultivar 'CTC9003' is less susceptible to Al stress because it showed better growth and physiological and morphoanatomy responses. In addition, the selection of plants that are more productive and tolerant to Al toxicity is a very important component for production strategies on acidic soils^52^. Our findings contribute to the current understanding of cultivar ‘CTC9003’ showed more acclimatization mechanisms to Si supplementation than cultivar ‘CTC9002’ under Al stress. Similar observation were reported in a tolerant maize cultivar to Al stress which was more productive under high Al concentrations conditions^53^. Similar results have been earlier reported in various plants species, such as wheat^54^, rice^33^ and barley^21,22^.

## Conclusions

The inclusion of Si in the nutrition of sugarcane seedlings under Al toxicity is beneficial as demonstrated in this study. Our data provide evidence that Si significantly increased growth and effectively mitigated the deleterious effects of Al. Si supplementation improved all the parameters evaluated such as root morphoanatomy, photosynthetic pigments, gas exchange, stomatal density, and growth. The cultivar ‘CTC9003’ was shown to be less sensitive to Al toxicity than the cultivar ‘CTC9002’, however, Si supplementation led to attenuation of the adverse impacts of Al toxicity on both cultivars. Taken together, our results clarified the role of Si in decreasing shoot Al concentration, restoring root development, and improving root and shoot growth under Al toxicity and provided scientific evidences for using silicon fertilizers to attenuate Al stress in acidic soils.

## Methods

### Growth conditions and vegetal material

The experiments were conducted in a glass greenhouse at São Paulo State University-UNESP (Campus of Jaboticabal, Brazil, geographical coordinates 21°15′22″ S and 48°18′58″ W), between February and September 2019. The seedlings of the sugarcane cultivars ‘CTC9002’ and ‘CTC9003’ used were from the Sugarcane Technology Center (CTC). The main characteristics of these sugarcane cultivars are drought tolerance, good suitability for mechanized planting, and higher sugar contents than other cultivars available on the market (UDOP, 2021). Seedlings were grown in an environment with natural light, 12/12 h photoperiod (light/dark), average day/night temperature of 26.7/18.2 °C, and relative humidity of 60% ± 15%. Buds of both cultivars were placed in plastic trays (1.7 dm^3^) filled with sterilized sand and irrigated with deionized water (without Si). Thirty days after emergence (DAE), the sugarcane seedlings were fixed on Styrofoam plates and root system was immersion in pots (3.8 dm^3^) filled with Clark's nutrient solution^55^, with pH = 4.5 (acidic conditions) or pH = 5.8 (normal conditions). Clark's nutrient solution used for plant growth contained the following composition (in mM): 2.6 Ca, 1.8 K, 0.6 Mg, 6.9 N-NO_3_, 0.9 N-NH_4_, 0.069 P, 0.5 S, 0.5 Cl, 0.038 Fe, 0.007 Mn, 0.019 B, 0.002 Zn, 0.0006 Mo, and 0.0005 Cu, with Si (2 mmol L^−1^) and Al (10, 15 and 20 mg L^−1^) varied among treatments. Clark’s nutrient solution started at 25% ionic strength and was added over 5 days to acclimate the plants. During the next 5 days, the Clark’s nutrient solution concentration was increased to 50%, and was changed to 100% ionic strength when the Al and Si treatments were started, remaining until the end of the experiment. The system was maintained under constant aeration of the solution by air bubbling supplied by a water pump (ACQ-001, BOYU-China).

### Experimental design

During the 45 days of the experimental growing period, two experiments were conducted in pots, distributed in randomized blocks, in a factorial arrangement (4 × 2) and four replicates/vessels for each treatment and for each sugarcane cultivar. The pots were divided into two plots; cv. ‘CTC9002’ and cv. ‘CTC9003’, and 32 pots were allocated to each plot/cultivar. Four treatments of Al, 0, 10, 15 and 20 mg L^−1^ as Al_2_(SO_4_)_3_·18H_2_O), were combined with the absence and presence (2.0 mmol L^−1^) of Si (as potassium silicate stabilized with sorbitol (113.4 g L^−1^ of Si and 18.9 g L^−1^ of K_2_O and pH 11.8). The experimental unit consisted in a pot (3.8 dm^3^) polyethylene pot filled with 3 m^3^ of Clark’s nutrient solution with one sugarcane seedling.

### Al and Si treatments

After transplanting, plants of both sugarcane cultivars were acclimated during 10 days in normal (pH = 5.8) and acidic (pH ≤ 4.5) Clark's nutrient solution. At this moment, Si treatments (2.0 mmol L^−1^, K_2_SiO_3_) was started via Clark’s nutrient solution to end of the experiment. Al treatments (0, 10, 15 and 20 mg L^−1^, Al_2_(SO_4_)_3_·18H_2_O) were started and maintained in acidic Clark’s nutrient solution in the corresponding pots, during the next 15 days. In the treatments that did not receive K_2_SiO_3_, K^+^ concentrations were balanced with potassium chloride (KCl). Plants without Al treatment were grown in neutral SN. During the growth period (35 days), the pH in neutral or acidic SN was adjusted daily with dilute hydrochloric acid (HCl), and both SN were changed every 5 days.

### Plant growth analysis

Thirty-five days after stress, the sugarcane seedlings were collected and the adhered residues were removed by washing with distilled water, detergent solution (0.2%), hydrochloric acid solution (0.1%), and finally twice with deionized water^56^. Leaf area was checked immediately after harvest using a leaf area meter (L-3100, Li-Cor, USA). Subsequently, the plants were separated into roots and aerial part, placed in paper bags and taken to the forced ventilation oven (M214Ai/BEL Analytics Equipment’s Ltd., Brazil) with temperature of 60 °C until reaching constant dry mass (DM). Then, the root and aboveground DM was immediately measured on a digital balance (Q31711-53/Quimis-Brazil).

### Determination of Al and Si concentration

The concentration of Al (g kg^−1^) in the tissues of both sugarcane cultivars (aerial part and roots) was carried out following the methodology of Wang and Wood^57^. Briefly, the dried samples were heated at 500 °C for 8 h and treated with HCl_2_. After filtration of the resulting solution, the total amount of Al was quantified by flame atomic absorption spectrophotometry (Corning 410, Essex, UK) at 324.7 nm. The Si concentration (g kg^−1^) in the aerial part was performed following a two-phase wet digestion procedure and Molybdenum Blue Colorimetry method as described by Kraska and Breitenbeck^58^. Young plant samples (0.1 g) were placed in 2 mL microtubes and moistened with 10 μL of octyl alcohol before the addition of 90 μL 30% H_2_O_2_. The tubes were tightly capped and placed in a convection oven set at 95 °C. After 30 min, 100 μL of 50% NaOH was added to the hot tubes, which were vortexed, tightly capped and returned to the oven and incubated at 95 °C for another 4 h. Immediately after digestion, 25 μL of 5 mM NH_4_ F was added to aid the formation of monosilicic acid. Si concentrations were determined using the ultraviolet spectrophotometer subsystem (SP-1105; Ningbo Hinotek Technology, Shanghai, China) at 410 nm.

### Determination of roots morphoanatomy

The morphoanatomical studies of the roots were carried out in the Plant Morphology Laboratory of the Sao Paulo State University—FCAV, Jaboticabal campus. To verify the anatomical alterations caused by the Al and Si addition in the root system of sugarcane, portions of approximately 5 mm from the main root apex were collected and stored in glass vials containing FAA solution (Formalin-aceto-alcohol: 90 mL of 50% ethanol, 5 mL of glacial acetic acid and 5 mL of 37% formaldehyde). For the preparation of permanent slides, the samples were dehydrated using the alcoholic series with tertiary butyl alcohol following Johansen's protocol^59^. The small apical portions of the main root were embedded in paraffin blocks, cross sectioned on a LEICA RM 2065 rotary microtome, and adhered to the histological slides with Meyer’s adhesive. The sections were contrasted with safranin, and for the final slide mounting, Canada balsam was used as adhesive for the coverslip. The evaluation occurred by measuring the overall root thickness and the thickness of the conducting vessels, and the material was observed and photographed on JENAVAL Carl Zeiss and Bel Photonics photomicroscopes.

### Determination of photosynthetic pigments

The determination of the plant pigments, chlorophylls (Chls *a* and Chls *b*), carotenoids (Cars) and anthocyanins (Anths) was performed in triplicate, using 50 mg of fresh leaf mass and the extraction followed the methodology of Lichtenthaler^60^. Chlorophylls and carotenoids extraction and Car 80% acetone were used and the extracts were kept for 48 h under refrigeration. For the extraction of Anths, 0.48 mL of methanol (99% v/v) acidified with HCl (99 mL methanol + 1 mL HCl) was used and stirred for 36 h at 60 rpm and 4 °C. Next, 0.36 mL of distilled H_2_O and 0.96 mL of chloroform were added and, proceeded to stir for 15 min, at 4 °C, at 5000 rpm. The absorbance readings of the solutions were performed in a Beckman spectrophotometer, model DU-640 at the wavelengths of 663 nm (Chls *a*), 647 nm (Chls *b*), 470 nm (Cars) and 535 nm (Anths).

### Determination of leaf gas exchange and stomatal density

The evaluation of stomatal conductance (*gs*) was performed with a diffusion porometer (Model AP4; Delta-T Devices Ltd.) at the end of the experiment between 9:00 am and 10:00 am. Transpiration rate (*E*), and net CO_2_ assimilation rate (*A*) were measured using a gas exchange system (LCpro, Analytical Development Co., Hoddeston, UK), assisted by a LED light source with a light intensity of 2000 µmol m^−2^ s^−1^. Stomatal density [number of adaxial stomata (ADS) and number of abaxial stomata (ABS)] was verified using the technique of cyanoacrylate-based adhesive agent printing of the adaxial and abaxial sides of the epidermis on microscope glass slides and counted using an optical microscope^61^.

### Statistical analysis

The data of the variables described above were submitted to the assumptions of normality and homogeneity of variance by the Shapiro–Wilk and Levenne tests, respectively. A Person’s correlation analysis, to verify the relationship between Si and Al concentrations were submitted^62^. Additionality, a multivariate analysis (two-way ANOVA) with Al and Si factors, as well as their interactions (Al × Si) were submitted. Then, when F values were significant (p < 0.05), the data were subjected to regression analysis, and equations were adjusted using the linear and polynomial models using GraphPad Prism v8.0 (GraphPad Inc., San Diego, CA, USA). For data interpretation, the equations with significance (p < 0.05) and the highest coefficients of determination (R^2^) were selected.. Furthermore, the means were compared using Tukey's HSD test (p < 0.05), when Fisher’s test (F) was significant (p < 0.05). All statistical analyses were performed in the software R (2019, Vienna, Austria).

### Compliance statement for experimental materials

Sugarcane is a widely distributed species in Brazil. Pot experiments were performed on the glass greenhouse at São Paulo State University-UNESP (Campus of Jaboticabal, Brazil, geographical coordinates 21°15′22″ S and 48°18′58″ W). Therefore, all operations comply with relevant institutional, national, and international guidelines and legislation.

### Supplementary Information

Supplementary Information.

## Data Availability

This manuscript includes all data generated or analyzed during this study. Other necessary data of this study are available with the corresponding author on reasonable request.
