# Supplementary material for: Silicon attenuates aluminum toxicity in sugarcane plants by modifying growth, roots morphoanatomy, photosynthetic pigments, and gas exchange parameters
Source: Sci Rep. 2024 Feb 27;14:4717. doi: 10.1038/s41598-024-53537-8 (PMC10899577; doi:10.1038/s41598-024-53537-8)
Supplement: Supplementary file 1 — Supplementary Information. [file 41598_2024_53537_MOESM1_ESM.pdf]

Supplementary Material

Silicon Attenuates Aluminum Toxicity in Sugarcane plants by Modifying Growth, Roots Morphoanatomy, Photosynthetic Pigments, and Gas Exchange Parameters

Gilmar da Silveira Sousa Junior[1*]; Alexander Hurtado Calero[2,3]; Rita de Cassia Alves[1]; Eduardo Custodio Gasparino[1]; Durvalina Maria Mathias dos Santos[1]

[1]Department of Biology Applied to Agriculture. School of Agricultural and Veterinarian Sciences. São Paulo State University (UNESP). Via de acesso Prof. Paulo Donato Castellane s/n, P. C. 14884-900, Jaboticabal, São Paulo, Brazil.
[2]Department of Agricultural Production Sciences - Soil and Fertilizer Sector. School of Agricultural and Veterinarian Sciences, São Paulo State University (UNESP). Via de acesso Prof. Paulo Donato Castellane s/n, P. C. 14884-900, Jaboticabal, São Paulo, Brazil.
[3]University of Sancti Spiritus "Jose Marti Perez" (UNISS). Comandante Fajardo, s/n, Olivos 2, P. C. 60100, Sancti Spiritus, Cuba.
*Corresponding author: E-mail: gilmar.ssjr@hotmail.com

Supporting information: 2 Figures and 5 Tables

Fig S1 Correlation analysis between [Al] and [Si] in the aerial part of sugarcane seedlings in both cultivars. [Al] under non-Si addition in 'CTC9002' a), [Al] under non-Si addition in 'CTC9003' b), [Al] under Si addition in 'CTC9002' c), and [Al] under Si addition in 'CTC9003' d),

Fig S2 Number of stomata adaxial in cv 'CTC9002' (a) and cv 'CTC9003' (b) and number of stomata abaxial in cv 'CTC9002' (c) and cv 'CTC9003' (d).

Table S1 Aerial dry mass of both sugarcane cultivars. ANOVA multifactorial with four Al concentrations, two Si levels and their interactions

Table S2 Root dry mass of both sugarcane cultivars. ANOVA multifactorial with four Al concentrations, two Si levels and their interactions

Table S3 Leaf area of both sugarcane cultivars. ANOVA multifactorial with four Al concentrations, two Si levels and their interactions

Table S4 Chlorophyll a content (Chls a) of both sugarcane cultivars. ANOVA multifactorial of with four Al concentration, two Si levels and their interactions

Table S5 Chlorophyll b content (Chls b) of both sugarcane cultivars. ANOVA multifactorial with four Al concentrations, two Si levels and their interactions

Table S6 Carotenoids (Cars) of both sugarcane cultivars. ANOVA multifactorial with four Al concentration, two Si levels and their interactions

Table S7 Anthocyanins (Anths) content of both sugarcane cultivars. ANOVA multifactorial of with four Al concentration, two Si levels and their interactions

Table S8 Stomatal conductance (*gs*) of both sugarcane cultivars. ANOVA multifactorial with four Al concentration, two Si levels and their interactions

Table S9 Transpiration rate (*E*) of both sugarcane cultivars. ANOVA multifactorial of with four Al concentration, two Si levels and their interactions

Table S10 CO_2 assimilation rate (*A*) of both sugarcane cultivars. ANOVA multifactorial with four Al concentrations, two Si levels and their interactions

Table S11 Number of adaxial stomata of both sugarcane cultivars ANOVA multifactorial with four Al concentrations, two Si levels and their interactions

Table S12 Number of abaxial stomata of both sugarcane cultivars. ANOVA multifactorial with four Al concentrations, two Si levels and their interactions

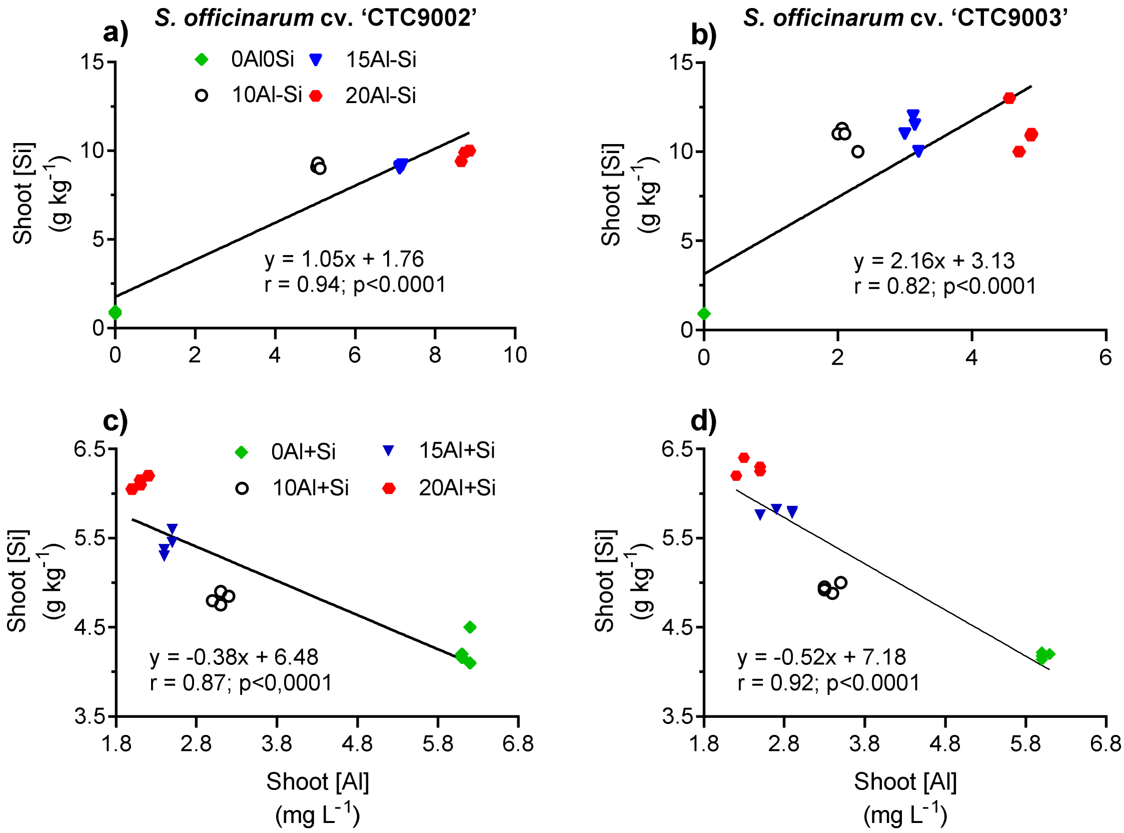

Fig S1 Correlation analysis between [Al] and [Si] in the aerial part of sugarcane seedlings in both cultivars. [Al] under non-Si addition in 'CTC9002' a), [Al] under non-Si addition in 'CTC9003' b), [Al] under Si addition in 'CTC9002' c), and [Al] under Si addition in 'CTC9003' d). Treatments: without Al stress and non-Si addition (0Al-Si); with Al stress (10 mg L^{-1}) and non-Si addition (10Al-Si); with Al stress (15 mg L^{-1}) and non-Si addition (15Al-Si) and Al stress (20 mg L^{-1}) and non-Si addition (20Al-Si); without Al stress and Si addition (0Al+Si); with Al stress (10 mg L^{-1}) and Si addition (2 mmol L-1) (10Al+Si); with Al stress (15 mg L^{-1}) and Si addition (2 mmol L^{-1}) (15Al+Si) and Al stress (20 mg L^{-1}) and Si addition (2 mmol L^{-1}) (20Al+Si)

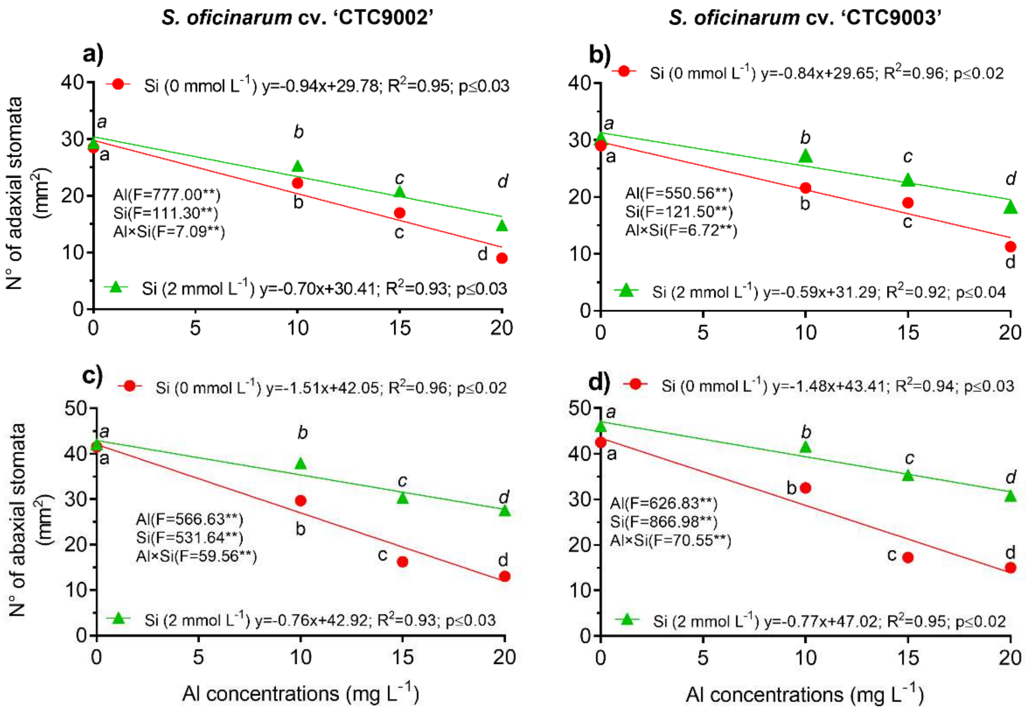

Fig S2 Number of adaxial stomata in cv 'CTC9002' (a) and in cv 'CTC9003' (b) and number of abaxial stomata in cv 'CTC9002' (c) and cv 'CTC9003' (d). Treatments: non-Al stress and Si addition (0Al+Si); Al stress (10 mg L^{-1}) and Si addition (2 mmol L^{-1}) (10Al+Si); with Al stress (15 mg L^{-1}) and Si addition (2 mmol L^{-1}) (15Al+Si) and Al stress (20 mg L^{-1}) and Si addition (2 mmol L^{-1}) (20Al+Si). Data are the means (n=4) ± standard deviation (SD). Different normal lowercase letters (e.g., a, b, c, d) indicate significant differences among Al concentrations under non-Si addition, and different italic lowercase letters (e.g., *a*, *b*, *c*) indicate significant differences among Al concentrations treatments under Si addition (2 mmol L^{-1}), according to the Tukey HSD test and F values from the ANOVA (p<0.05)

Table S1 Aerial dry mass of both sugarcane cultivars. ANOVA multifactorial with four Al concentrations, two Si levels and their interactions

**Aerial dry mass (g)**					
**'CTC9002'**					
**Variation causes**	**DF**	**SS**	**MS**	**F (DFn, DFd)**	**P value**
Al concentrations	3	130.77	43.59	F (3, 21) = 197.35**	0.0001
Si supplementation	1	19.96	19.95	F (1, 21) = 90.34**	0.0001
Interaction (Al × Si)	3	10.75	3.65	F (3, 21) = 16.53**	0.0001
Residual	21	4.64	0.22		
**'CTC9003'**					
**Variation causes**	**DF**	**SS**	**MS**	**F (DFn, DFd)**	**P value**
Al concentrations	3	112.52	37.51	F (3, 21) = 297.53**	0.0001
Si supplementation	1	21.13	21.13	F (1, 21) = 167.58**	0.0001
Interaction (Al × Si)	3	8.65	2.88	F (3, 21) = 22.87**	0.0001
Residual	21	2.64	0.12		

Note: *: indicates significant at $p < 0.05$ and **: indicates significant at $p < 0.01$.

Table S2 Root dry mass of both sugarcane cultivars. ANOVA multifactorial with four Al concentrations, two Si levels and their interactions

Root dry mass (g)					
**'CTC9002'**					
**Variation causes**	**DF**	**SS**	**MS**	**F (DFn, DFd)**	**P value**
Al concentrations	3	32.17	10.72	F (3, 21) = 45.03**	0.0001
Si supplementation	1	2.45	2.45	F (1, 21) = 10.30**	0.0042
Interaction (Al × Si)	3	1.22	0.41	F (3, 21) = 11.71**	0.0002
Residual	21	5.00	0.24		

'CTC9003'					
**Variation causes**	**DF**	**SS**	**MS**	**F (DFn, DFd)**	**P value**
Al concentrations	3	29.29	9.76	F (3, 21) = 121.63**	0.0001
Si supplementation	1	5.37	5.37	F (1, 21) = 66.91**	0.0001
Interaction (Al × Si)	3	1.47	0.49	F (3, 21) = 6.11**	0.0037
Residual	21	1.69	0.08		

Note: *: indicates significant at $p < 0.05$ and **: indicates significant at $p < 0.01$.

Table S3 Leaf area of both sugarcane cultivars. ANOVA multifactorial with four Al concentrations, two Si levels and their interactions

Leaf area (cm^2)					
**'CTC9002'**					
**Variation causes**	**DF**	**SS**	**MS**	**F (DFn, DFd)**	**P value**
Al concentrations	3	1059751.0	353250.3	F (3, 21) = 4469.50**	0.0001
Si supplementation	1	68635.13	68635.13	F (1, 21) = 868.41**	0.0001
Interaction (Al × Si)	3	23040.38	7680.13	F (3, 21) = 97.17**	0.0001
Residual	21	1659.75	79.04		
**'CTC9003'**					
**Variation causes**	**DF**	**SS**	**MS**	**F (DFn, DFd)**	**P value**
Al concentrations	3	963767.7	321255.7	F (3, 21) = 8200.72**	0.0001
Si supplementation	1	76342.78	76342.78	F (1, 21) = 1948.81**	0.0001
Interaction (Al × Si)	3	27033.34	9011.11	F (3, 21) = 230.03**	0.0001
Residual	21	822.66	39.17		

Note: *: indicates significant at $p < 0.05$ and **: indicates significant at $p < 0.01$.

Table S4 Chlorophyll a content (Chls a) of both sugarcane cultivars. ANOVA multifactorial of with four Al concentration, two Si levels and their interactions

**Chls a**					
**'CTC9002'**					
**Variation causes**	**DF**	**SS**	**MS**	**F (DFn, DFd)**	**P value**
Al concentrations	3	20.21	6.74	F (3, 21) = 862.94**	0.0001
Si supplementation	1	4.81	4.81	F (1, 21) = 616.49**	0.0001
Interaction (Al × Si)	3	4.66	1.55	F (3, 21) = 199.06**	0.0001
Residual	21	0.16	0.01		

**'CTC9003'**					
**Variation causes**	**DF**	**SS**	**MS**	**F (DFn, DFd)**	**P value**
Al concentrations	3	10.90	3.63	F (3, 21) = 339.16**	0.0001
Si supplementation	1	5.19	5.19	F (1, 21) = 484.74**	0.0001
Interaction (Al × Si)	3	4.52	1.51	F (3, 21) = 140.53**	0.0001
Residual	21	0.22	0.01		

Note: *: indicates significant at $p < 0.05$ and **: indicates significant at $p < 0.01$.

Table S5 Chlorophyll b content (Chls b) of both sugarcane cultivars. ANOVA multifactorial with four Al concentrations, two Si levels and their interactions

**Chls b**					
**'CTC9002'**					
**Variation causes**	**DF**	**SS**	**MS**	**F (DFn, DFd)**	**P value**
Al concentrations	3	21.98	7.33	F (3, 21) = 2227.68**	0.0001
Si supplementation	1	6.04	6.04	F (1, 21) = 1835.93**	0.0001
Interaction (Al × Si)	3	2.29	0.76	F (3, 21) = 231.95**	0.0001
Residual	21	0.07	0.003		

**'CTC9003'**					
**Variation causes**	**DF**	**SS**	**MS**	**F (DFn, DFd)**	**P value**
Al concentrations	3	15.66	5.22	F (3, 21) = 1865.43**	0.0001
Si supplementation	1	4.35	4.35	F (1, 21) = 1555.34**	0.0001
Interaction (Al × Si)	3	2.02	0.67	F (3, 21) = 240.23**	0.0001
Residual	21	0.06	0.002		

Note: *: indicates significant at $p < 0.05$ and **: indicates significant at $p < 0.01$.

Table S6 Carotenoids (Cars) of both sugarcane cultivars. ANOVA multifactorial with four Al concentration, two Si levels and their interactions

Cars					
**'CTC9002'**					
**Variation causes**	**DF**	**SS**	**MS**	**F (DFn, DFd)**	**P value**
Al concentrations	3	11.93	3.98	F (3, 21) = 710.72**	0.0001
Si supplementation	1	2.76	2.76	F (1, 21) = 493.50**	0.0001
Interaction (Al × Si)	3	1.33	0.44	F (3, 21) = 79.46**	0.0001
Residual	21	0.12	0.01		

'CTC9003'					
**Variation causes**	**DF**	**SS**	**MS**	**F (DFn, DFd)**	**P value**
Al concentrations	3	10.83	3.61	F (3, 21) = 412.62**	0.0001
Si supplementation	1	2.00	2.00	F (1, 21) = 228.57**	0.0001
Interaction (Al × Si)	3	1.62	0.54	F (3, 21) = 61.81**	0.0001
Residual	21	0.18	0.01		

Note: *: indicates significant at $p < 0.05$ and **: indicates significant at $p < 0.01$.

Table S7 Anthocyanins (Anths) content of both sugarcane cultivars. ANOVA multifactorial of with four Al concentration, two Si levels and their interactions

**Anths**					
**'CTC9002'**					
**Variation causes**	**DF**	**SS**	**MS**	**F (DFn, DFd)**	**P value**
Al concentrations	3	0.002	0.0008	F (3, 21) = 523.64**	0.0001
Si supplementation	1	0.0002	0.0002	F (1, 21) = 96.35**	0.0001
Interaction (Al × Si)	3	0.00007	0.00002	F (3, 21) = 14.92**	0.0001
Residual	21	0.00003	0.000002		

**'CTC9003'**					
**Variation causes**	**DF**	**SS**	**MS**	**F (DFn, DFd)**	**P value**
Al concentrations	3	0.002	0.0005	F (3, 21) = 598.52**	0.0001
Si supplementation	1	0.0001	0.0001	F (1, 21) = 128.57**	0.0001
Interaction (Al × Si)	3	0.00005	0.00002	F (3, 21) = 18.95**	0.0001
Residual	21	0.00002	0.000001		

Note: *: indicates significant at $p < 0.05$ and **: indicates significant at $p < 0.01$.

Table S8 Stomatal conductance (*gs*) of both sugarcane cultivars. ANOVA multifactorial with four Al concentration, two Si levels and their interactions

gs					
**'CTC9002'**					
**Variation causes**	**DF**	**SS**	**MS**	**F (DFn, DFd)**	**P value**
Al concentrations	3	1.72	0.57	F (3, 21) = 1644.15**	0.0001
Si supplementation	1	0.01	0.01	F (1, 21) = 35.73**	0.0001
Interaction (Al × Si)	3	0.02	0.005	F (3, 21) = 15.08**	0.0001
Residual	21	0.007	0.0003		

**'CTC9003'**					
**Variation causes**	**DF**	**SS**	**MS**	**F (DFn, DFd)**	**P value**
Al concentrations	3	2.76	0.92	F (3, 21) = 529.16**	0.0001
Si supplementation	1	0.09	0.09	F (1, 21) = 53.21**	0.0001
Interaction (Al × Si)	3	0.10	0.03	F (3, 21) = 18.63**	0.0001
Residual	21	0.03	0.002		

Note: *: indicates significant at $p < 0.05$ and **: indicates significant at $p < 0.01$.

Table S9 Transpiration rate (*E*) of both sugarcane cultivars. ANOVA multifactorial of with four Al concentration, two Si levels and their interactions

E					
**'CTC9002'**					
**Variation causes**	**DF**	**SS**	**MS**	**F (DFn, DFd)**	**P value**
Al concentrations	3	2.95	0.98	F (3, 21) = 149.21**	0.0001
Si supplementation	1	0.14	0.14	F (1, 21) = 21.15**	0.0001
Interaction (Al × Si)	3	0.02	0.006	F (3, 21) = 6.94**	0.0001
Residual	21	0.14	0.007		
**'CTC9003'**					
**Variation causes**	**DF**	**SS**	**MS**	**F (DFn, DFd)**	**P value**
Al concentrations	3	2.54	0.85	F (3, 21) = 119.88**	0.0001
Si supplementation	1	0.17	0.17	F (1, 21) = 23.64**	0.0001
Interaction (Al × Si)	3	0.04	0.01	F (3, 21) = 11.89**	0.0001
Residual	21	0.15	0.007		

Note: *: indicates significant at $p < 0.05$ and **: indicates significant at $p < 0.01$.

Table S10 CO_2 assimilation rate (*A*) of both sugarcane cultivars. ANOVA multifactorial with four Al concentrations, two Si levels and their interactions

A					
**'CTC9002'**					
**Variation causes**	**DF**	**SS**	**MS**	**F (DFn, DFd)**	**P value**
Al concentrations	3	52.87	17.62	F (3, 21) = 4146.28**	0.0001
Si supplementation	1	15.10	15.10	F (1, 21) = 3551.86**	0.0001
Interaction (Al × Si)	3	7.35	2.45	F (3, 21) = 576.59**	0.0001
Residual	21	0.09	0.004		
**'CTC9003'**					
**Variation causes**	**DF**	**SS**	**MS**	**F (DFn, DFd)**	**P value**
Al concentrations	3	40.67	13.56	F (3, 21) = 878.47**	0.0001
Si supplementation	1	14.72	14.72	F (1, 21) = 953.59**	0.0001
Interaction (Al × Si)	3	6.74	2.24	F (3, 21) = 145.50**	0.0001
Residual	21	0.32	0.02		

Note: *: indicates significant at $p < 0.05$ and **: indicates significant at $p < 0.01$.

Table S11 Number of adaxial stomata of both sugarcane cultivars ANOVA multifactorial with four Al concentrations, two Si levels and their interactions

Number of stomata adaxial					
**'CTC9002'**					
**Variation causes**	**DF**	**SS**	**MS**	**F (DFn, DFd)**	**P value**
Al concentrations	3	1571.34	523.78	F (3, 21) = 777.00**	0.0001
Si supplementation	1	75.03	75.03	F (1, 21) = 111.30**	0.0001
Interaction (Al × Si)	3	14.34	4.78	F (3, 21) = 7.09**	0.0001
Residual	21	14.16	0.67		
**'CTC9003'**					
**Variation causes**	**DF**	**SS**	**MS**	**F (DFn, DFd)**	**P value**
Al concentrations	3	1238.75	412.92	F (3, 21) = 550.56**	0.0001
Si supplementation	1	91.13	91.13	F (1, 21) = 121.50**	0.0001
Interaction (Al × Si)	3	15.13	5.04	F (3, 21) = 6.72**	0.0024
Residual	21	15.75	0.75		

Note: *: indicates significant at $p < 0.05$ and **: indicates significant at $p < 0.01$.

Table S12 Number of abaxial stomata of both sugarcane cultivars. ANOVA multifactorial with four Al concentrations, two Si levels and their interactions

Number of stomata abaxial					
**'CTC9002'**					
**Variation causes**	**DF**	**SS**	**MS**	**F (DFn, DFd)**	**P value**
Al concentrations	3	2400.59	800.20	F (3, 21) = 566.63**	0.0001
Si supplementation	1	750.78	750.78	F (1, 21) = 531.64**	0.0001
Interaction (Al × Si)	3	252.34	84.11	F (3, 21) = 59.56**	0.0001
Residual	21	29.66	1.41		
**'CTC9003'**					
**Variation causes**	**DF**	**SS**	**MS**	**F (DFn, DFd)**	**P value**
Al concentrations	3	2319.84	773.28	F (3, 21) = 626.83**	0.0001
Si supplementation	1	1069.53	1069.53	F (1, 21) = 866.98**	0.0001
Interaction (Al × Si)	3	261.09	87.03	F (3, 21) = 70.55**	0.0001
Residual	21	25.91	1.23		

Note: *: indicates significant at $p < 0.05$ and **: indicates significant at $p < 0.01$.
